# Complex Regional Pain Syndrome Developing After a Coral Snake Bite: A Case Report

**DOI:** 10.7759/cureus.9787

**Published:** 2020-08-16

**Authors:** Elis M Cruz Salcedo, Anamarys Blanco, Justin Reed

**Affiliations:** 1 Internal Medicine, Ocala Regional Medical Center/University of Central Florida College of Medicine, Ocala, USA

**Keywords:** complex regional pain syndrome, snake bite, gabapentin, budapest criteria, physical therapy

## Abstract

Complex regional pain syndrome (CRPS) usually occurs after an inciting injury. Poor understanding of pathophysiology, management, and disease awareness has led to misdiagnosis of this condition. We report a rare case of a 69-year-old male who developed CRPS following a Florida Coral snake bite on his right foot. Initially, it was misdiagnosed as recurrent cellulitis; however, he developed chronic right lower extremity (RLE) pain with worsening flares associated with right leg swelling and erythema. Examination was remarkable for nonpitting edema, erythema, and severe tenderness to light touch of the RLE, all symptoms that highly supported the diagnosis of CRPS. Treatment was initiated and consisted of physical therapy in addition to gabapentin which resulted in marked improvement. CRPS remains a challenging diagnosis due to lack of gold standard test and can be easily misdiagnosed. Clinical evaluation applying Budapest criteria can aid with diagnosis and should be routinely used for all patients with suspected CRPS.

## Introduction

Complex regional pain syndrome (CRPS), formerly known as reflex sympathetic dystrophy, is characterized by a continuing, spontaneous and/or evoked, regional pain that is disproportionate in time or degree to the usual course of pain after trauma or other injury [1]. Although the current understanding of its pathophysiology is a growing field, studies have hypothesized that inflammation, central and peripheral sensitization, autoimmunity, genetic and psychological factors play an important role in the development of this syndrome [2]. Most common inciting factors are distal extremity fracture and/or surgery [3], with other less recognized injuries to be potential triggers, such as snakebites. 

Complex regional pain syndrome was first described in 1994, and it is hypothesized that phospholipase A2, metalloproteinase, inflammatory substances such as interleukins and tumor necrosis factors, along with toxicities from snake venom are implicated in local tissue effects and may play a role in the development of CRPS [4-5]. Increased sensitivity to catecholamines due to sympathetic denervation and upregulation of peripheral receptors have also been proposed as pathogenic mechanisms [6]. The estimated incidence of CRPS is 5.5-26.2 cases per 100,000 people per year, but the occurrence of CRPS after a snake bite is very rare with only four cases reported worldwide and none reported in the United States to our knowledge [2, 5-8]. Here we describe a rare case of CRPS that developed in a male patient after a snake bite on his right foot that was previously misdiagnosed as recurrent cellulitis.

## Case presentation

**Figure 1:**
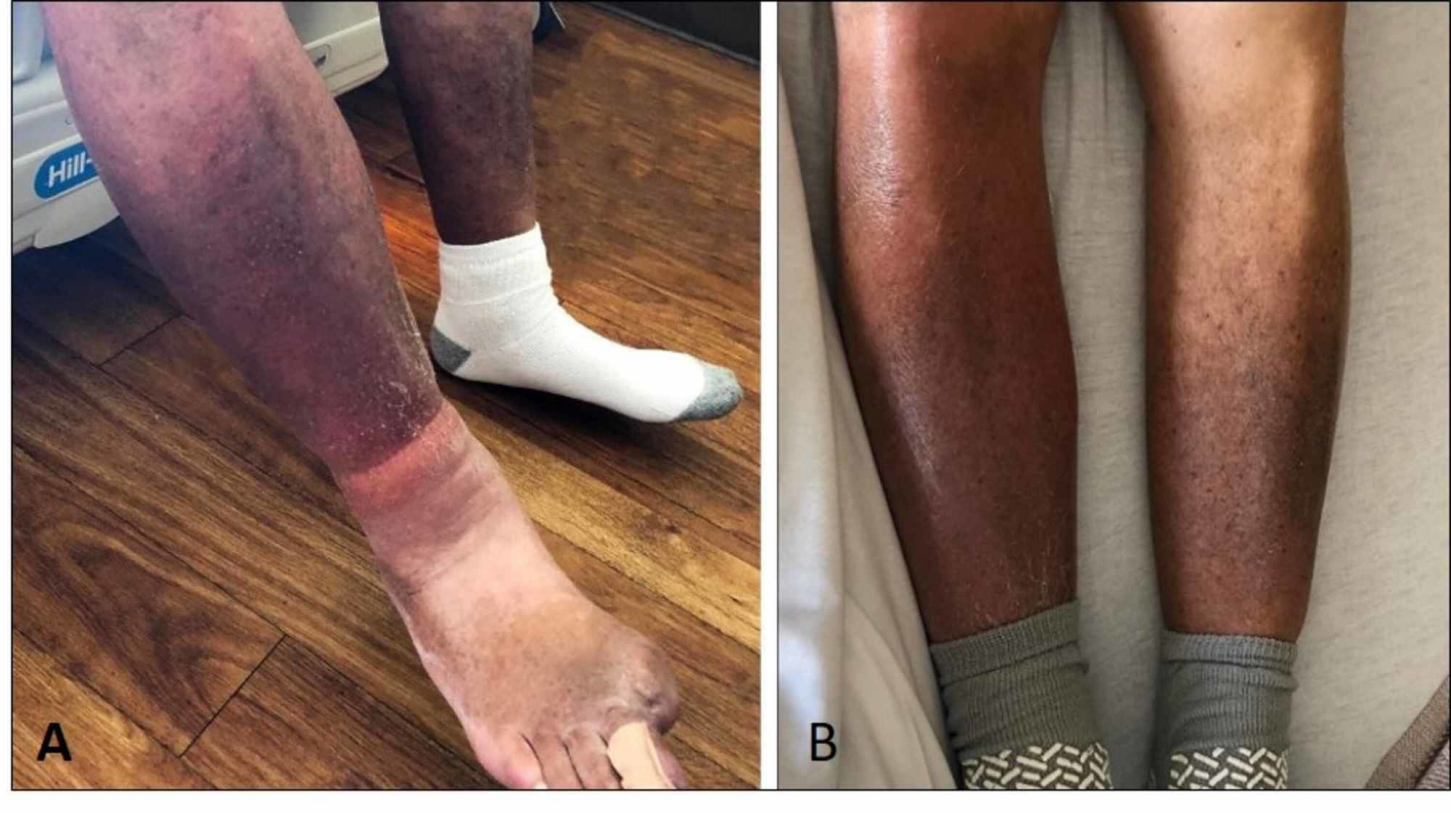
Pictures demonstrating edema and color asymmetry. A. Depicts edema and discoloration of right lower extremity. B. Demonstrates color and size asymmetry of the right lower extremity when compared with the left side.

**Figure 2:**
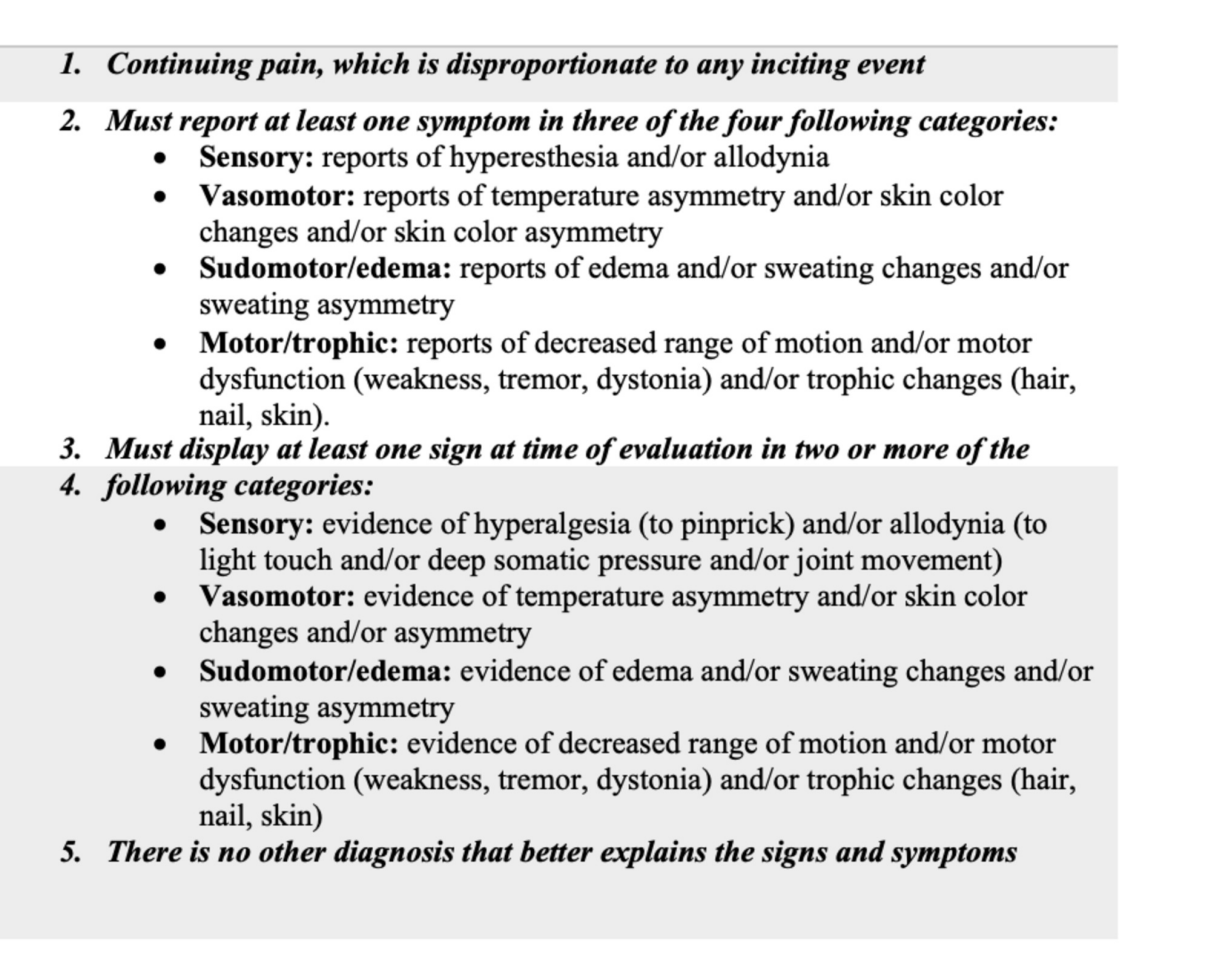
Budapest clinical diagnosis criteria for CRPS. CRPS, complex regional pain syndrome

A 69-year-old male with a history of coronary artery disease, cirrhosis secondary to nonalcoholic steatohepatitis, chronic kidney disease, and diabetes type 2 presented with complaints of worsening right lower extremity (RLE) pain, 10 out of 10 in severity, radiating to his abdomen, associated with right leg swelling and erythema for one week, with no fever or chills. A detailed history revealed that three years preceding presentation the patient was bitten by a Florida Coral snake (*Micrurus fulvium*) on his right hallux, with no antivenom given due to unavailability, requiring amputation of the right great toe due to secondary infection at the site of the bite. Since then, the patient has had chronic RLE pain with worsening flares that were treated as cellulitis with no improvement of symptoms. The patient was afebrile with a heart rate of 104 beats per minute. Examination was remarkable for nonpitting edema, erythema, warmth, extreme tenderness to light touch and decreased range of motion of RLE, with chronic skin color changes noted bilaterally (Figure 1). Left lower extremity examination was unremarkable, except chronic venous stasis discoloration changes. Laboratory studies showed leukocyte count of 7,900 cells/mL with normal differential, and no neutrophilic left shift. RLE ultrasound doppler was negative for deep venous thrombosis and CT revealed nonspecific subcutaneous soft tissue edema and osteoarthritic changes in the subtalar joint. The provisional diagnosis of CRPS was made using Budapest criteria, supported by allodynia, skin color asymmetry, and edema, not better explained by another diagnosis (Figure 2) [9]. The patient initiated physical therapy and started gabapentin 200 mg orally twice a day with marked improvement of symptoms. The patient was referred to pain specialist for further evaluation.

 

## Discussion

Complex regional pain syndrome incited by a snake bite is infrequent, as demonstrated by a medical literature research yielding only four reported cases worldwide. To the best of our knowledge, this is the first CRPS case reported after snakebite in the United States [5-8]. One known hypothesis suggests that snake venom can play a role in causation or exaggeration of CRPS added to the role of local inflammation, pain, and tissue damage [5]. Antivenom use has failed to be effective against local inflammation suggestive of the multifactorial nature of the disease [5]. The pathophysiology and severity of envenomation differs depending on the snake species. Pit viper venom, the first species reported in the literature with an association of CRPS, causes local tissue necrosis and coagulopathy, while Vipera berus venom, as reported in a Norway case, is also known to evoke pain and localized inflammatory effects [7]. Coral snake venom, as in our case, gains systemic access through the lymphatic veins and uses competitive inhibition of the muscarinic acetylcholine receptors at the neuromuscular junction [10]. Review of the literature demonstrates a complex pathophysiology with ill-defined etiology and predisposing factors making CRPS difficult to diagnose and opens up new venues for research on treatment options.

Complex regional pain syndrome remains a challenging diagnosis due to lack of a gold standard test, and evidence-based information. As evidenced in this case, this may lead to misdiagnosis, delay in treatment, and exposure to inappropriate treatments such as antibiotics, contributing to antibiotic resistance and prolongation of pain. International Association for the Study of Pain (IASP) diagnostic criteria for CRPS was established at a consensus in 1994 and includes the presence of an initiating noxious event or cause of immobilization; continuing pain, allodynia, or hyperalgesia in which the pain is disproportionate to inciting event; evidence at some point of edema, changes in skin blood flow, or abnormal sudomotor activity in the region of pain; and diagnosis is excluded by the existence of other conditions that would otherwise account for pain and dysfunction [1]. Weaknesses of these criteria include poor specificity (0.36), diagnosis made only by historical symptoms reported, and failure to include motor/trophic symptoms, which may lead to overdiagnosis [1]. There is a proposed modified diagnostic criterion, known as Budapest criteria that adds four categories, sensory, vasomotor, sudomotor/edema, and motor/trophic, requiring at least one symptom within three out of four categories, and at least one sign at the time of evaluation in two or more categories for the clinical diagnosis to be made with improvement of specificity (Figure 2) [1]. Validation studies have been performed with the proposed Budapest criteria against the IASP criteria, results revealing similar sensitivities (0.99 vs 1.00, respectively), but greater specificity (0.79 vs 0.41, respectively) [4]. Therefore, clinical evaluation applying Budapest criteria should be routinely used for all patients with suspected CRPS as it can aid with diagnosis given the lack of a definitive diagnostic test [3].

Differential diagnosis in our case included necrotizing fasciitis, peripheral vascular disease (PVD), deep vein thrombosis, peripheral neuropathy, and infection of the skin or bone although less likely as the patient was afebrile with a normal leukocyte count. As per the patient, he was also evaluated in the outpatient setting for PVD, with no intervention and no findings suggestive of occlusion. RLE ultrasound doppler was negative for deep venous thrombosis and CT revealed nonspecific subcutaneous soft tissue edema and osteoarthritic changes in the subtalar joint without evidence of gas, abscess, or fluid collection. Hence, a fourth criterion was met as no other diagnosis explained better signs and symptoms of our patient. 

Treatment for CRPS is mainly symptomatic with a combination of physical therapy, psychological therapy, neuropathic pain medications, anti-inflammatories and interventional procedures, but management continues to be poorly defined and benefits have to be weighed against side effects when deciding treatment modalities. Additional evidence-based information about effectiveness is needed, as most decisions are based on prior studies showing efficacy of these modalities on neuropathic pain [3]. A randomized double-blind placebo-controlled crossover study showed evidence of pain relief and improvement of sensory deficit in favor of gabapentin use initially with no significant pain reducing effect overall [11]. In our case, the patient was initiated on physical therapy and started on gabapentin 200 mg orally twice a day with marked improvement of symptoms during hospitalization, but due to the patient being lost to follow up no further evaluation was done to assess long-term improvement.

As our patient presented to our institution three years after a Florida Coral snake bite, details of the history may be forgotten as we rely mostly on historical data and symptoms reported by patient and family members. At this point, lack of evidence of outpatient data limited our understanding of the studies already done. Given clinical scenario, history, findings, and improvement of symptoms over the course of his hospital stay pointed to the diagnosis of CRPS. Unfortunately, as mentioned above, the patient was lost to follow up affecting continuity of care and optimization of his condition.

## Conclusions

Complex regional pain syndrome remains a challenging diagnosis with ill-defined pathophysiology. It is commonly misdiagnosed delaying treatment and exposing patients to inappropriate treatments. As demonstrated by medical literature, CRPS incited by a snake bite is infrequent. Given the lack of gold standard test, the use of Budapest criteria may increase specificity of the diagnosis and should be implemented as part of the clinical evaluation. This rare case further contributes to the current literature, aiming to increase awareness of CRPS clinical recognition and snakebites as inciting events to the development of this disorder. 
